# Fibroblastic reticular cells provide a supportive niche for lymph node–resident macrophages

**DOI:** 10.1002/eji.202250355

**Published:** 2023-07-12

**Authors:** Joshua D'Rozario, Konstantin Knoblich, Mechthild Lütge, Christian Pérez Shibayama, Hung‐Wei Cheng, Yannick O. Alexandre, David Roberts, Joana Campos, Emma E. Dutton, Muath Suliman, Alice E. Denton, Shannon J. Turley, Richard L. Boyd, Scott N. Mueller, Burkhard Ludewig, Tracy S.P. Heng, Anne L. Fletcher

**Affiliations:** ^1^ Department of Biochemistry and Molecular Biology and Monash Biomedicine Discovery Institute Monash University Clayton Australia; ^2^ Department of Anatomy and Developmental Biology Monash Biomedicine Discovery Institute Monash University Clayton Australia; ^3^ Institute of Immunology and Immunotherapy University of Birmingham Birmingham UK; ^4^ Institute of Immunobiology Kantonsspital St. Gallen St. Gallen Switzerland; ^5^ Department of Microbiology and Immunology The Peter Doherty Institute for Infection and Immunity The University of Melbourne VIC Melbourne Australia; ^6^ Institute of Inflammation and Ageing University of Birmingham Birmingham UK; ^7^ Department of Immunology and Inflammation Imperial College London London UK; ^8^ Department of Cancer Immunology Genentech Inc. South San Francisco CA USA; ^9^ Cartherics Pty Ltd Hudson Institute for Medical Research Clayton Australia; ^10^ ARC Training Centre for Cell and Tissue Engineering Technologies Monash University Clayton Australia

**Keywords:** CSF1, Fibroblastic reticular cells, Human lymph nodes, Lymph node stromal cells, Macrophages

## Abstract

The lymph node (LN) is home to resident macrophage populations that are essential for immune function and homeostasis, but key factors controlling this niche are undefined. Here, we show that fibroblastic reticular cells (FRCs) are an essential component of the LN macrophage niche. Genetic ablation of FRCs caused rapid loss of macrophages and monocytes from LNs across two in vivo models. Macrophages co‐localized with FRCs in human LNs, and murine single‐cell RNA‐sequencing revealed that FRC subsets broadly expressed master macrophage regulator CSF1. Functional assays containing purified FRCs and monocytes showed that CSF1R signaling was sufficient to support macrophage development. These effects were conserved between mouse and human systems. These data indicate an important role for FRCs in maintaining the LN parenchymal macrophage niche.

## Graphical abstract

Lymph node fibroblastic reticular cells (FRCs) support monocyte and macrophage survival and differentiation. When FRCs were depleted in vivo, myeloid cells disappeared. In vitro, both mouse and human FRCs supported the survival and differentiation of nonclassical monocytes and M2 macrophages, in part by signaling through CSF1R (figure created using Biorender).

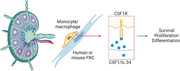

## Introduction

In LNs, stromal cell communication with leukocytes is key to the initiation of a healthy immune response and eventual pathogen control [1, 2]. Fibroblastic reticular cells (FRCs) are the most prevalent nonhematopoietic cell type in LNs. Together with sinusoidal vascular elements, FRCs create the structural scaffolding on which leukocytes migrate and interact, including the T cell and dendritic cell (DC)‐rich paracortex, the cortical B‐cell follicles, and the medullary plasma cell niche [1, 3, 4]. From this unique position at the coalface of the immune response, FRCs have evolved an immune‐specialized role in regulating the survival, interaction, migration and function of T cells, B cells, DCs, plasma cells, and innate lymphoid cells [1, 2, 3, 4, 5, 6, 7, 8].

The importance of FRCs in adaptive immunity is well established, and emerging evidence suggests that they also play a critical role in innate immunity. Specialized Gremlin1^+^ subsets of fibroblasts engage in multifaceted crosstalk with DCs in lymphoid tissues, and in some systems fibroblasts and macrophages are capable of co‐operative support through mutual provision of growth factors [6, 9, 10, 11]. Mouse FRCs also respond to viral and bacterial ligands [12, 13, 14, 15], regulate viral clearance through expression of the innate immunological sensing adaptor MyD88 [16], and in nonclassical secondary lymphoid organs, FRC‐dependent MyD88 signaling steers B‐cell responses via TNF‐dependent interactions with inflammatory monocytes [17] and promotes GC responses to immunization in aged mice [12].

Despite the pivotal role of macrophages as managers of immune homeostasis and drivers of humoral and anti‐viral immunity, our understanding of macrophage biology in LNs is still evolving. Macrophages are found in every FRC niche [18, 19], but key factors controlling their development, function, and localization in LNs remain unclear. LN macrophages are categorized by the niches they occupy. Sinus‐lining macrophages, incorporating subcapsular and medullary sinus‐lining subsets, are relatively well‐studied as highly phagocytic “flypaper” macrophages bathed in lymph and capable of engulfing incoming antigen for efficient presentation to other cell types [19]. Subcapsular sinus macrophages capture particles or immune complexes for direct transfer to follicular B cells and are important for limiting viral spread [20, 21, 22, 23, 24]. They can present antigen to naïve T cells, but their ability to internalize and process antigen is lower than medullary sinus macrophages, which are highly efficient at pathogen and apoptotic cell clearance [24]. Depletion of both subsets in mice reduced anti‐tumor immunity through a reduction in CD8^+^ T‐cell activation [25]. Mesenchymal lymphoid tissue organizer cells and marginal reticular cells utilize RANKL to support the development of subcapsular and medullary CD169^+^ sinusoidal macrophages, but not other macrophage subsets [26].

Less well studied are the macrophages found in parenchymal and FRC‐rich regions of LNs. Medullary cord macrophages regulate medullary plasma cell maturation and survival [4, 27], while in the T‐cell paracortical zone, a rich, previously misidentified network of immunosuppressive macrophages play a unique role in immune homeostasis through potent efferocytosis of apoptotic T cells [18, 19]. Both macrophage populations strongly co‐localize with their local FRC network [4, 19].

Here, we identified an essential support system provided by FRCs to macrophages. Using two complementary mouse models, we demonstrate that depletion of FRCs drives a rapid loss of myeloid cells in LNs. We show that FRCs and macrophages co‐localize, FRC subsets broadly express CSF1, and FRCs are capable of regulating macrophage differentiation and survival through CSF1R signaling to myeloid lineage cells. These data show that FRCs are likely to regulate the LN macrophage niche.

## Results

### FRC ablation diminishes myeloid cell lineages within LNs

**Figure 1:**
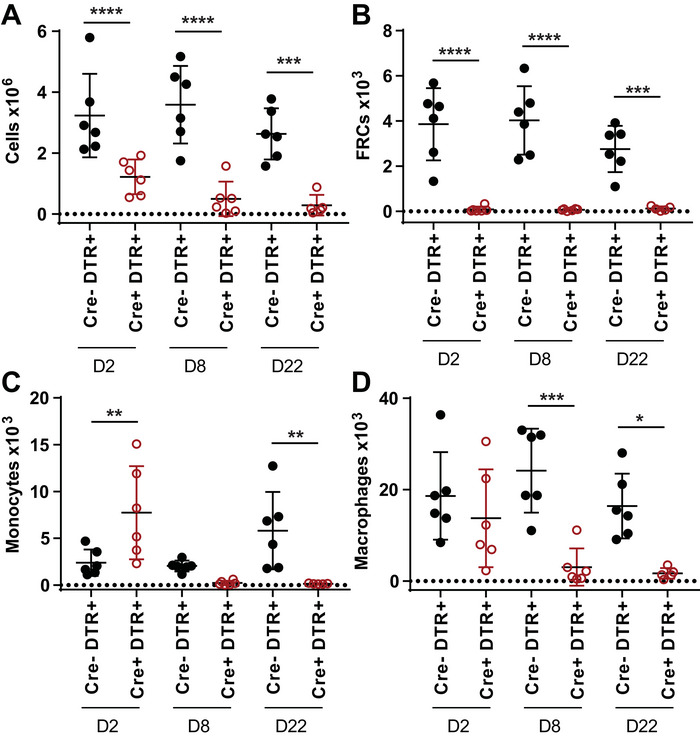
**In vivo depletion of FRCs induces rapid loss of myeloid‐lineage cells**. CCL19‐DTR mice or DTR‐expressing Cre‐negative littermate controls were treated with diphtheria toxin, and brachial lymph nodes were harvested and analyzed by flow cytometry at 2, 8, or 22 days after treatment ceased. (**A)** Total LN cellularity. (**B)** FRC numbers, gated as CD45^‐^CD31^‐^gp38^+^ cells. (**C)** Monocyte numbers, gated as B220^‐^NK1.1^‐^CD3e^‐^Ly6C^high^CD11b^+^. (**D)** Macrophage numbers, gated as B220^‐^NK1.1^‐^CD3e^‐^Ly6C^+/‐^F480^+^. All graphs depict mean + SDM from N = 4–5 mice per group from two independent experiments. Statistical significance was assessed using a one‐way ANOVA with Sidak's multiple comparisons test: *p < 0.05, **p < 0.01, ***p < 0.001, ****p < 0.0001.

To examine the impact of removing FRCs on major myeloid cell types in the LN, we used two mouse models of FRC depletion. First, in vivo depletion of FRCs was achieved by crossing a CCL19‐Cre strain with a Rosa26‐diphtheria toxin receptor strain (CCL19‐DTR), as described [3]. In this mouse model, diphtheria toxin (DTx) treatment efficiently depletes T‐zone and marginal zone FRCs within LNs and 50–75% of medullary FRCs [4]. Compared with littermate controls, 48 h after DTx treatment, LNs of CCL19‐Cre^+^DTR^+^ mice underwent significant and prolonged involution, involving overall reduced cellularity that did not recover for the duration of the study (3 weeks) (Fig. 1A). FRCs were strongly ablated at day 2 posttreatment and did not recover within the study duration (Fig. 1B, Supporting information Fig. S1A). Markers expressed by major monocyte and macrophage populations showed that monocytes underwent an initial influx into the LN prior to loss (Fig. 1C) in line with influx described in other models of inflammation [28], and macrophages similarly showed a delayed loss, with no effect at day 2 and a significant depletion at day 8 that did not recover by day 22 (Fig. 1D). These data show that monocyte and macrophage populations were not sustained in the absence of FRCs.

In a separate FRC depletion model, where FRCs express DTR under the control of fibroblast activation protein (FAP; DM2 mice [29]), we observed a similar effect of FRC depletion on monocyte and macrophage numbers (Supporting information Fig. S1B–D) compared with treated control mice. LNs harvested 2 days after the cessation of DTx treatment showed that FRC ablation led to a significant reduction in monocytes and macrophages.

These findings demonstrated that FRCs are necessary to maintain myeloid lineage cells within secondary lymphoid organs. Since FRCs maintain many cell types in LNs, we wanted to assess whether FRCs were likely to provide direct myeloid cell support.

### T zone macrophages colocalize with FRCs in secondary lymphoid organs

**Figure 2:**
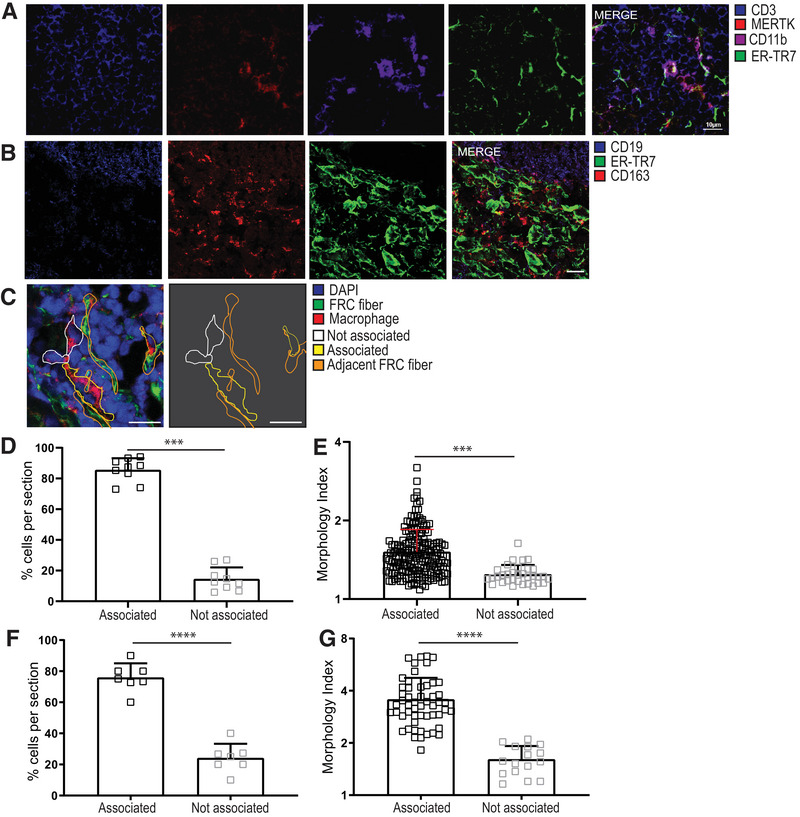
**T**‐**zone macrophages colocalize with FRCs**. (**A)** Mouse lymph node (LN) immunofluorescence depicting MERTK^+^CD11b^+^ macrophages and ERTR7^+^ FRCs within the T‐zone region of the LN (CD3^+^), representative of *n* = 3 mice. Scale bar = 10 µm. (**B)** Human tonsil immunofluorescence depicting association of CD163^+^ macrophages with ER‐TR7^+^ FRCs in an area of paracortex adjacent to a B‐cell follicle. Scale bar = 50 µm. Imaging represents *n* = 3 human donors. (**C)** Macrophages with a clear DAPI^+^ nucleus had their perimeter outlined and were designated as either associated (yellow outline) or not associated (white outline) with ERTR7^+^ FRC reticular fibers based on the proportion of colocalized perimeter. Human tonsil section shown, representative of analysis for both mouse and human tissues. Scale bar = 25 µm. (**D)** The proportion of macrophages per mouse LN section designated as associated or not associated with ERTR7^+^ reticular fibers. Each datapoint represents an individual tissue section. Data depict *n* = 3 mice. (**E)** Morphology index (perimeter^2^/(4π*area)) was calculated for each MERTK+ macrophage observable in a clear cross‐section with visible DAPI^+^ nucleus. Each datapoint represents an individual macrophage, and aggregate data are shown from 224 cells, from *n* = 3 mice and eight tissue sections. Scale bar = 10 µm. (**F** and **G)** Macrophages were designated as associated or not associated with ERTR7^+^ reticular fibers, as described in (C). (**G)** Morphology index was calculated for each macrophage as described in (C). Data are shown from 69 cells, from *N* = 3 human donors and seven tissue sections. Graphs depict mean + SDM. All statistics shown are Mann–Whitney *U* test; ***p < 0.001, ****p < 0.0001.

To first identify whether FRCs and macrophages were in direct contact, we examined their relationship in secondary lymphoid organs using immunohistology. Some data on this exists: in mice, MERTK^+^ macrophages are known to interact with FRCs in the T‐cell zone [18] and accordingly, in mouse LNs, we also observed macrophages colocalizing with ER‐TR7^+^ fibers (Fig. 2A). Macrophages in mice exist in every FRC niche [18, 19]. Human histology similarly showed FRCs and CD163^+^ macrophages in close proximity adjacent to a B‐cell follicle (Fig. 2B). CD163 is a scavenger receptor that denotes anti‐inflammatory, chronically inflamed, tumor associated, and wound‐healing M2 macrophage responses, and CD163^+^ macrophages are found in perifollicular, interfollicular marginal and medullary areas of human LNs [30, 31, 32].

We noted that in both species, macrophages in contact with FRC fibers appeared elongated, fitting with previous observations for T cells, B cells, and DCs when engaged with FRC fibers [33]. We wanted to quantify these interactions to test the hypothesis of physical association between the cell types, so we calculated how much of the macrophage perimeter was in direct contact with an FRC fiber, taking 10% colocalization as a cutoff to exclude minor incidental contact, and scoring the proportion of macrophages associated or not associated (Fig. 2C). We road‐tested this metric using mouse LN sections, since that cell–cell association is previously published [18], and found that macrophages were significantly more likely to be associated (>10% of perimeter in contact) with an FRC fiber than not associated (<10%) (Fig. 2D). As further support, using a calculation previously used to describe DC/FRC interactions [34], we found that macrophages that were associated with FRCs showed significantly higher morphology index, where a higher number shows a greater perimeter to area ratio, supportive of the elongation seen when other hematopoietic cells attach to FRCs (Fig. 2E). We then tested the association in human tonsils and found that macrophages co‐localized with FRCs (Fig. 2F) and showed significantly higher morphology index when associated with FRCs (Fig. 2G). These data provide evidence for a physical relationship between macrophages and the FRC network that is conserved across human and mouse LNs.

### FRCs express immunoregulatory factors for myeloid cell maturation, migration, and function

**Figure 3:**
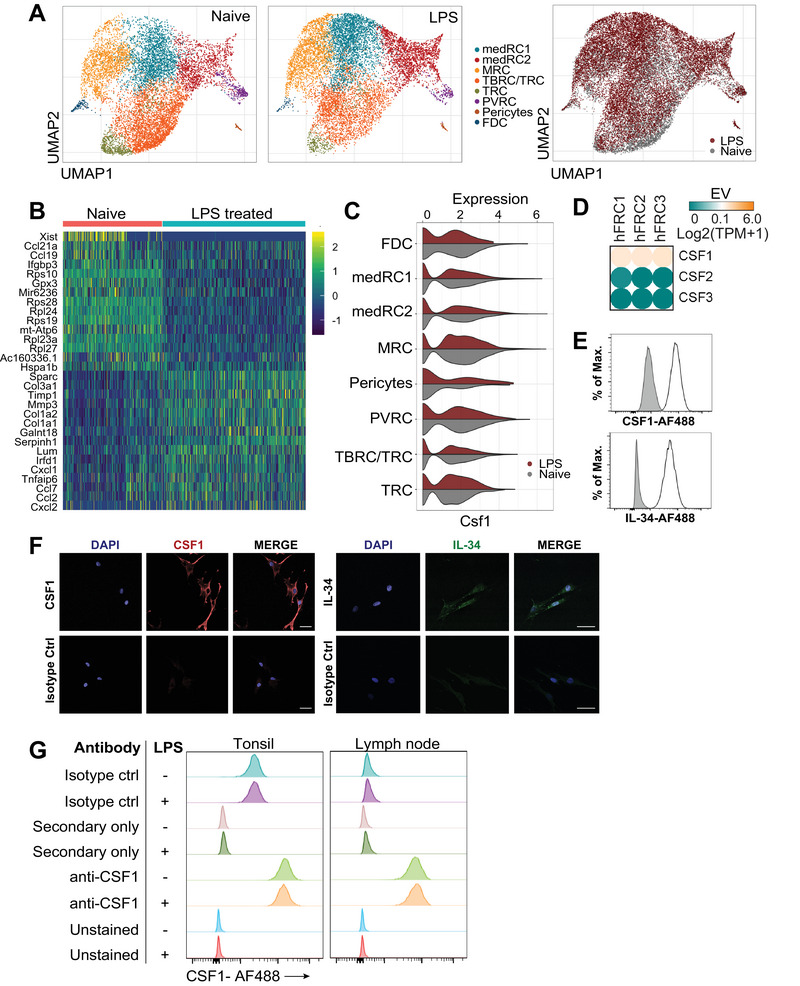
**Mouse and human FRCs express genes relevant to myeloid cell maturation, recruitment, and function. A**. EYFP^+^ reticular cells were isolated from brachial lymph nodes from Ccl19 Cre R26R‐EYFP mice, which were either treatment‐naïve (*n* = 2) or immunized with OVA/LPS (*n* = 2) and analyzed 3 days later. scRNA‐seq was performed on EYFP^+^ cells. UMAP of EYFP+ LN reticular cells colored by the assigned subsets, with or without treatment. UMAP of merged data from LPS‐treated or naive EYFP^+^ LN reticular cells is shown on the rightmost plot. (**B)** Top 15 differentially expressed genes between reticular cell clusters from naïve and LPS‐treated mice. (**C)** Violin plots showing expression of Csf1 by FRC subsets in treated or naïve mice. (**D)** RNA‐Seq data from culture‐expanded human tonsil FRCs harvested at passage 3. *n* = 3 unrelated human donors are depicted. EV: expression value; hFRC: human fibroblastic reticular cells; TPM: transcripts per million. Below the expression threshold is shown as green, genes at the expression threshold are shown as white, transitioning to orange for expression, generated using Morpheus software (Broad Institute). (**E)** Human FRCs at passage 3 were analyzed for expression of IL‐34 and CSF1 protein using flow cytometry. Isotype control antibody staining shown in gray, representative of two donors. (**F)** Immunofluorescent images of human FRCs stained for IL‐34 and CSF1 protein compared to isotype control antibodies. Scale bars are 50 µm. Data depict two donors. (**G)** Human LN or tonsil FRCs from two donors were cultured with or without LPS as described in (C). Flow cytometry for CSF1 is shown compared with staining controls. Data representative of two independent experiments.

To determine which FRC subsets expressed factors relevant to myeloid cell support, we investigated the global transcriptional profile of FRCs in CCL19‐Cre x R26R‐EYFP mice, treated with or without LPS as a likely stimulator of an innate immune transcriptional response. EYFP^+^ reticular cells from the LNs were sorted for single‐cell RNA‐Seq analysis. Eight fibroblastic clusters were identified and validated via expression of expected markers (Fig. 3A; Supporting information Fig. S2A–C) [8, 35]. The subsets identified comprised T‐zone reticular cells (TRCs), follicular FDCs, pericytes, perivascular reticular cells, marginal reticular cells, T‐B zone reticular cells (TBRC/TRC), and two subsets of medullary reticular cells (medRC1 and medRC2), and all subsets were present in treated and untreated mice (Fig. 3A, Supporting information Fig. S2A and B).

Next, we compared LPS‐treated versus treatment‐naïve mice and identified the 30 most differentially expressed genes (15 upregulated with treatment, 15 downregulated) across all clusters. LPS treatment was associated with upregulated expression of myeloid cell‐attracting chemokines including Cxcl1, Cxcl2, and Ccl2. Conversely, expression of homeostatic chemokines Ccl19 and Ccl21a was downregulated, as well as genes encoding ribosomal proteins (Fig. 3B). KEGG (Kyoto encyclopedia of genes and genomes) pathway analysis (*p* < 0.05, false discovery rate and Benjamini–Hochberg < 0.05) showed that LPS treatment drove significant overrepresentation of genes related to innate immune function, including TNF, chemokine, cytokine, and NOD‐like receptor signaling (Supporting information Fig. S2D).

Based on their prominent roles in myeloid cell regulation, we examined Ccl2 and Csf1 expression. These were robustly expressed across all mouse FRC subsets. In the single‐cell dataset, Ccl2 was upregulated with LPS (Supporting information Fig. S2E), and Csf1 did not change, remaining strongly expressed (Fig. 3C). These findings were validated at mRNA and protein levels in human FRCs from three donors (Fig. 3D–G, Supporting information Fig. S2E and F).

The proliferation, differentiation, and survival of many macrophage populations is regulated by CSF1, which acts via autocrine or paracrine signaling through its receptor, CSF1R [36, 37]. IL‐34, which is another, more recently discovered CSF1R ligand, was also expressed in single‐cell RNA‐seq analysis of freshly isolated human Gremlin1^+^ fibroblasts [6] and we identified expression in our cultured human FRCs (Fig. 3E and F). These data strongly suggest that FRCs generate CSF1R ligands.

The known biological importance of CSF1R signaling, as a fundamental regulator of macrophage development, fitted with our findings of macrophage depletion from in vivo models of FRC deletion, leading us to test whether FRCs could directly support myeloid cell development or differentiation via provision of CSF1R ligands.

### FRCs can regulate the survival and differentiation of monocytes via signaling to CSF1R

To investigate the effects of FRC‐derived CSF1R signaling on myeloid cells, we first performed co‐culture assays with mouse BM cells as a source of myeloid precursors including monocytes. Monocytes can be defined as classical (CD11b^+^Ly6C^hi^ in mouse, CD14^+^CD16^−^ in human) or non‐classical (CD11b^+^Ly6C^lo^ in mouse, CD14^−/lo^CD16^+^ in human) based on their ability to perform pro‐ or anti‐inflammatory functions [38]. Macrophages can be identified in bulk (in mice, CD11b^+^F4/80^+^), but exhibit effects on a continuum from strongly pro‐inflammatory (often denoted M1; human phenotype CD64^+^HLA‐DR^+^) to strongly suppressive (M2; human phenotype CD206^+^CD64^−^) [39, 40]. Their differentiation depends on the cues given by the local tissue micro‐environment [37].

**Figure 4:**
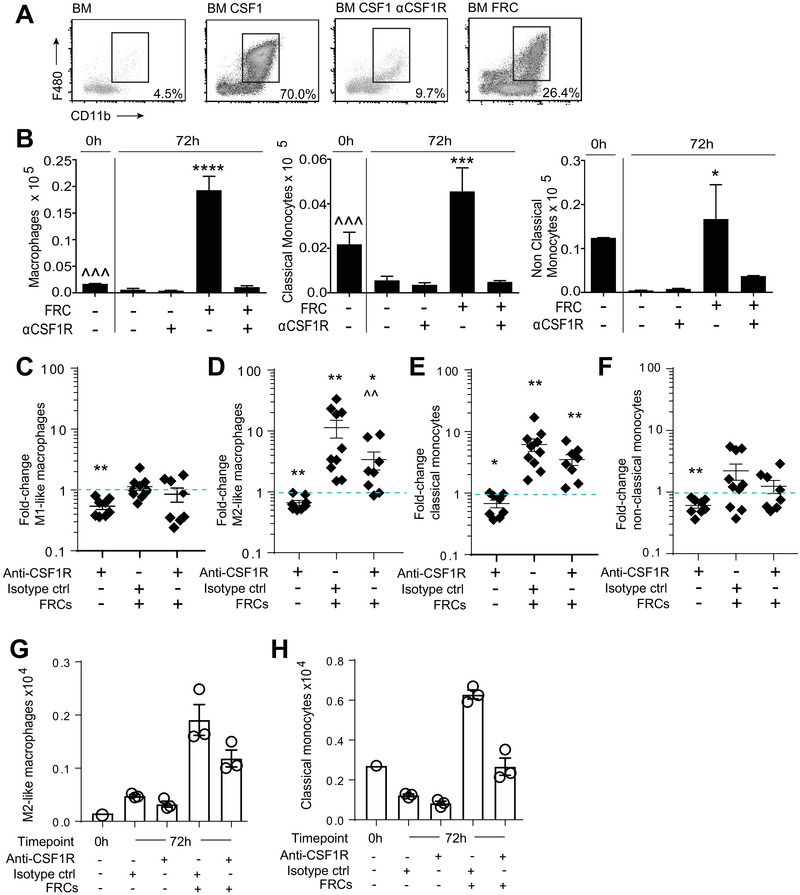
**Fibroblastic reticular cells support monocyte differentiation via CSF1R signaling**. (**A** and **B)** 1 × 10^6^ mouse BN cells were co‐cultured with 2 × 10^5^ mouse FRCs with or without recombinant CSF1, or anti‐CSF1R blocking antibody as shown. (**A)** Representative flow cytometry profiles for F4/80 and CD11b after 72 h of culture. Macrophages were gated on CD45^+^ GR‐1^‐^. Monocytes were gated as non‐classical (GR‐1^‐^ F480^lo^ CD11b^+^ Ly6C^lo^) or classical (GR‐1^‐^ F480^lo^ CD11b^+^ Ly6C^hi^). (**B)** Macrophage and monocyte numbers were quantified using flow cytometry at either 0 h (input analysis) or after 72 h of culture. Mean ± SEM shown. Data depict *n* = 4 mice and are representative of three independent experiments. *p < 0.05, ***p < 0.001, ****p < 0.0001 versus untreated 72 h; ^^^*p* < 0.001 0 h versus FRC‐treated, one‐way ANOVA with Tukey's posttest. Mean ± SEM shown. (**C–H)** 4 × 10^5^ human peripheral blood mononuclear cells (PBMCs) were phenotyped immediately after isolation (0H) or incubated with or without CSF1R blocking antibody, isotype control antibody, or 2 × 10^4^ human tonsil‐derived FRCs. After 72 h of culture, cells were quantified and analyzed via flow cytometry. The fold change in cell number, compared to untreated controls (normalized to 1, shown as dotted blue line), is depicted for *n* = 2 FRC and *n*=3 PBMC donors from three independent experiments: (**C)** M1‐like macrophages (CD64^+^HLA‐DR^+^CD206^‐^), (**D)** M2‐like macrophages (CD206^+^HLA‐DR^+^CD64^‐^), (**E)** Classical monocytes (CD16^‐^CD14^+^), (**F)** nonclassical monocytes (CD16^+^CD14^‐/lo^). Absolute cell number from one representative donor (one experiment) is depicted for (**G)** M2‐like macrophages and (**H)** classical monocytes. All subsets were gated negative for CD3, CD19, CD56, CD135 before gating as described above. Graphs depict mean ± SEM. *p < 0.05, **p < 0.01; Wilcoxon rank test with a ratio paired *t* test. Star depicts significance versus untreated group (normalized to 1); chevron depicts significance versus FRC + isotype control group. FRCs: fibroblastic reticular cells. CSF1: colony stimulating factor 1; CSF1R: CSF1 receptor; αCSF1R: anti‐CSF1R blocking antibody.

After 72 h in the presence of recombinant CSF1, as expected, mouse BM cells readily differentiated to CD11b^+^F4/80^+^ macrophages, and this was inhibited by the addition of a CSF1R blocking antibody (Fig. 4A, Supporting information Fig. S3A and B).

Notably, the addition of FRCs to mouse BM cells, without exogenous CSF1, was sufficient to yield an expansion of macrophages and classical monocytes over the culture period, and this was dependent on CSF1R signaling (Fig. 4A and B). Absolute numbers of macrophages and classical monocytes significantly increased after plating, suggesting that FRCs actively fostered the proliferation and/or differentiation of these cells. FRCs were also able to maintain numbers of nonclassical monocytes, which otherwise underwent attrition over the culture period (Fig. 4B).

Based on these results, we then explored the effects of human FRCs and FRC‐derived CSF1R ligands on human monocyte and macrophage phenotypes. We used a co‐culture system with PBMCs from healthy donors as a monocyte source and examined numbers and phenotypes of major monocyte and macrophage subsets after 3 days with or without FRCs and CSF1R blockade (Supporting information Fig. S3C).

Human FRCs did not alter M1‐phenotype macrophages (Fig. 4C), but provided a strong differentiation stimulus for M2‐phenotype macrophages (Fig. 4D). This effect of FRCs was significantly reduced in the presence of a CSF1R blocking antibody. FRCs also drove an average eightfold increase in CD16^−^CD14^+^ classical monocytes, however this was not mediated through CSF1R signaling, since CSFR1 blockade did not prevent the increase (Fig. 4E). This suggested the presence of undescribed CSF1R‐independent mechanisms of FRC support. Human FRCs did not alter numbers of CD16^+^CD14^−^ nonclassical monocytes (Fig. 4F). These data were broadly concordant with mouse results.

Fold‐change increases in M2 macrophages and classical monocytes with FRC co‐culture represented an absolute increase in cell numbers over 72 h of culture, showing that FRCs support an active increase in these cells rather than simply fostering survival (Fig. 4G and H).

Together, these data show that mouse and human FRCs are likely to play necessary and sufficient roles in macrophage biology within LNs and tonsil. FRCs colocalized with and were necessary for macrophage survival in vivo, and signaling provided by FRCs to the CSF1R was alone sufficient to drive M2 macrophage differentiation and survival in a reductionist system. Additional undefined FRC‐derived signals were involved in driving the FRC‐mediated increase classical monocytes.

## Discussion

FRCs form the structural highway on which leukocytes interact. FRCs have been shown to facilitate deletional tolerance [13], antigen presentation [41, 42], and lymphocyte and DC homing [43, 44]. FRCs promote T cell, B cell, plasma cell, and innate lymphoid cell survival [3, 4, 16, 44], regulate T‐cell activation in mice and humans [1], and are known to respond to LPS [12, 14, 16, 17, 45,]. Recently, targeted deletion of type I IFN receptor from FRCs revealed a role in infection‐driven monocyte and neutrophil accumulation and recruitment, revealing an important biological imperative to understand FRC‐innate immune cell interactions within LNs and secondary lymphoid organs [46].

The presence of resident macrophages within LNs has been long reported [19, 31], and lymphatic endothelial cells (LECs) are known to maintain or drive development of lymphatic‐adjacent subcapsular sinus and medullary sinus macrophages through provision of CSF1 and RANK [26, 47]. However, while monocytes and macrophages are also confirmed in FRC‐rich zones [4, 18, 28, 48], the cells and factors supporting their residence remain undefined. Here, we sought to define immunoregulatory factors involved in the intimate relationship between FRCs and myeloid cells.

Immunofluorescence microscopy of human tonsil and mouse LNs revealed that macrophages colocalize with FRC fibers and elongate when aligned with FRC fibers. Our transcriptomic analysis of mouse and human FRCs built on previous findings and showed strongly conserved expression of genes relevant to innate immunity, particularly the expression of CSF1, CCL2, IL‐6, IL‐8/CXCL8, and CXCL12, all of which have well‐established roles in the maturation, function, adhesion, and/or chemoattraction of myeloid cells [49, 50, 51, 52, 53].

To explore expression of myeloid‐supporting factors by FRCs in vivo, in steady‐state and inflamed conditions, we performed single‐cell RNA‐seq data and identified and validated eight reticular cell clusters from CCL19‐Cre x R26R‐EYFP mice, supporting previous single‐cell RNA‐seq analyses, and demonstrating the niche specific heterogeneity of these cell types [6, 35]. Differences between T‐B zone reticular cells and TRCs are likely to be gradually acquired, with genes being expressed along a continuum ([8, 46]), and in this study, we identified a large population of TBRC likely to represent a mixed population of TBRCs and TRCs, labeled as TBRC/TRC. Characterisation of clusters was based on the expression of a combination of known marker genes and expression pattern of unbiased calculated marker genes. This approach is important as the expression pattern of single genes can vary between tissue site and cellular state, even between LNs from distinct sampling sites ([54, 55]). This can only be resolved by cross‐organ studies that will identify more conserved marker genes in the future.

Csf1 was robustly expressed across all mouse FRC types, driving the central hypothesis that FRCs may support myeloid cells through the provision of CSF1R signaling. Co‐culture experiments accordingly showed that FRCs were able to support the differentiation and survival of M2‐like macrophages via signaling through CSF1R. A study, accepted while this paper was in review, similarly showed that mouse FRCs drive development of M2 macrophages in vitro through provision of Csf1 [56] and our findings now support and extend this finding to human M2 macrophages.

CSF1 expression was not altered after LPS treatment of mice or of human FRCs, and it is attractive to speculate that FRCs promote macrophage polarization toward a regenerative and repair state, and away from an inflammatory state, as FRCs dampen innate immune‐driven inflammation in murine sepsis [45, 57]. While mouse and human FRCs exhibit some clear molecular differences in regulation of T‐cell activation [7], our results showed that both the effects of FRCs on macrophages and monocytes and a CSF1R‐dependent signaling mechanism are strongly conserved between mice and humans.

The importance of an intact FRC network for macrophage maintenance was demonstrated using in vivo depletion of FRCs in two mouse models, CCL19‐DTR and FAP‐DTR. Both models showed a rapid loss of resident macrophages. The lack of recovery was not unexpected, as alterations in FRC networks can take several weeks to months to resolve [58, 59]. The molecular mechanisms are unknown. Certainly both models induce a loss of various cell types, including T cells, B cells, and DCs that are dependent on FRCs for recruitment, function, and survival [3, 5, 29], but there is nonetheless a clear dependence on FRCs, direct or indirect, for maintenance of monocyte and macrophage numbers within LNs.

The biology of human FRCs is still under open exploration, and it is unclear which subsets of FRCs are best represented through in vitro culture. Recent findings [4, 35, 60] have shown that mouse FRC subsets include a distinct medullary population that regulates plasma cell function via production of APRIL and IL‐6. Medullary macrophage populations are reportedly CSF1‐independent in mice [50, 53, 61]. These sinusoidal myeloid populations have recently been shown to respond to RANKL signaling from marginal reticular fibroblasts and LECs [26], with LEC‐derived CSF1 shown to regulate sinus‐lining (subcapsular and medullary) macrophage populations [47]. T‐zone macrophages were also relatively recently defined [18], and whether they specifically rely on CSF1 is not yet known. All fibroblast subsets in adult mice, including T‐zone subsets, expressed either CSF1 or IL‐34 in mice and we demonstrated protein expression of these ligands on the whole FRC population when grown in culture. IL‐34 and CSF1 both bind the CSF1R and possess similar ability to promote macrophage differentiation, though their roles diverge beyond this point, driving differential cytokine secretion by macrophages [62, 63]. Any temporal requirement for CSF1 signaling by FRCs in the development or maintenance of macrophages is also yet to be tested but may be relevant; for example, in mice, deletion of lymphatic endothelial RANK signaling impaired the development of normal numbers of subcapsular and medullary sinusoidal macrophages at timepoints from embryonic day E18.5 through to 2 weeks postnatal, while deletion from 4 weeks old had no effect [26]. FRC‐adjacent macrophages in the T zone of LNs can derive postnatally from BM monocytes [18] so there may be multiple opportunities for the described relationships to take effect.

Using complementary mouse and human studies, our data showed that macrophages interact intimately with FRCs in vivo and ultimately rely on FRCs for survival. Importantly, in vivo depletion experiments revealed that an intact stromal network is critically important to the maintenance of macrophages and monocytes. These findings show that both mouse and human FRCs provide microenvironmental support to macrophages and monocytes.

## Materials and methods

### Human tissues

Palatine tonsils were obtained from consenting donors from the National Disease Research Interchange resource center or Human Biomaterials Resource Centre (HBRC), Birmingham (HTA license 12358, 15/NW/0079), under project approval number REC/RG/HBRC/12‐071. Primary human FRC lines originating from inguinal, axillary, or mesenteric LNs were previously described [7]. FRCs were functionally identical regardless of source, and data from multiple lines is provided throughout the analysis. FRCs were confirmed monocultures with expected characteristics [7]. Human blood from healthy consenting donors was obtained under MUHREC approval, project number 11939. All tissues were obtained and utilized in accordance with institutional guidelines and according to the principles expressed in the Declaration of Helsinki.

### Experimental Animals

C57BL6J mice were obtained from Monash Animal Services and housed at the Animal Research Laboratories at Monash University, Clayton. CCL19‐Cre mice [64] were crossed with Rosa26‐iDTR mice and maintained at the Peter Doherty Institute, The University of Melbourne. DM2 mice expressing the DTR under the regulatory elements of the murine *Fap* gene [29, 65] were housed at the University of Birmingham Biomedical Services Unit. For single cell RNA‐Seq, BAC‐transgenic C57BL/6N‐Tg (Ccl19‐Cre)489Biat (Ccl19‐Cre) mice [64] were previously described. All mice used were between 4 and 10 weeks of age. Experiments were performed in accordance with federal and cantonal guidelines (Tierschutzgesetz) under permission numbers SG07/19 following review and approval by the Cantonal Veterinary Office (St. Gallen, Switzerland). All mice were specific pathogen free and cared for in accordance with institutional guidelines. All experiments received approval from relevant institutional ethics committees.

### LN digestion and FRC purification

Murine axillary, mesenteric and brachial LNs harvested from 5 to 10 euthanized mice were digested and cultured according to published protocols [66]. Cell suspensions were seeded at approximately 2900 cells/cm^2^ in antibiotic‐free conditions. FRCs were purified using MACS (Miltenyi Biotec) as described [66], using anti‐mouse CD45 and CD31 magnetic beads. Cells were quantified using a hemocytometer and assessed for viability using trypan blue. Viability and purity were routinely >92%.

### Mice and LPS administration

Ccl19‐Cre x R26R‐EYFP mice were subcutaneously injected in one flank with 5 mg of LPS from *Escherichia coli* (Sigma) together with 100 mg Ovalbumin grade VI (Sigma). Mice were euthanized at day 3 postadministration, and brachial LNs were removed for further LN stromal cell isolation and single‐cell RNA‐seq analysis.

### Droplet‐based single cell RNA‐seq analysis

Stromal cells from enzymatically digested brachial LNs were depleted of CD45^+^ hematopoietic cells and TER119^+^ erythrocytes using MACS microbeads (Miltenyi Biotec) as described [64]. EYFP^+^CD31^−^CD45^−^ reticular cells were sorted from two biological replicates of LPS‐treated and two biological replicates of naïve controls and processed on a 10x chromium (10X Genomics) to generate cDNA libraries following the recommended protocol for the Chromium Single Cell 3’ Reagent Kit (v3 Chemistry). Libraries were sequenced on an Illumina NovaSeq 6000 at the Functional Genomic Center Zurich. Sequencing files were pre‐processed using CellRanger (v3.0.2) [67] with Ensembl GRCm38.94 release as reference and damaged cells or duplicates were removed using scater R/Bioconductor package (v1.11.2) [68] running in R v3.6.0. Cells were excluded if they had high or low UMI (unique molecular identifier) counts or total detected genes or high mitochondrial gene content (>2.5 median absolute deviations above the median across all cells). In addition, cells expressing any of Top2a, Mki67, Pclaf, or Cenpf genes were excluded as proliferating cells, leaving 10 019 cells from naïve LNs and 14 427 cells from LPS‐treated mice.

Downstream analysis was performed using the Seurat package (v3.1.1) [69] running in R v3.6.1, including data normalization, scaling, dimensional reduction with principal component analysis  and uniform manifold approximation and projection (UMAP), and graph‐based clustering. Clusters were characterized based on marker genes and conditions were compared based on differentially expressed genes inferred from Wilcoxon rank test as implemented in the FindMarkers function (Seurat v3.1.1) [69]. Functional differences between conditions were summarized based on a gene set enrichment analysis. All genes were ranked based on a S/N statistic calculated on normalized expression values. Resulting ranked gene lists were used as input for GSEA‐Preranked (v7.0.3) in a GenePattern notebook [70] with gene sets from the mSigDB (v7.0) C2 collection (accession number: E‐MTAB‐10908).

Bulk RNA‐Seq data from cultured human FRCs was previously described [7], and the heatmap was generated using Morpheus matrix visualization software (Broad Institute).

### In vivo FRC ablation

CCL19‐Cre x Rosa26‐iDTR (CCL19‐DTR) mice and Cre‐negative Rosa26‐iDTR^+^ controls received two injections of DTx at 10 ng/g of body weight, 24 h apart. Skin draining LNs were used for analysis. DTR FAP^+^ DM2 mice received 25 ng/g DTx (List Biological Laboratories) i.p. on days 0, 2, and 4 and were euthanized on day 6. LNs (axillary, brachial, inguinal, and mesenteric) were used for analysis.

### Luminex bead assay

Human FRCs were stimulated with 1 µg/mL LPS (Serotype O111:B4; Sigma Aldrich) for 24 h. Where stated, wells were pre‐cultured for 2 h with 10 mg/mL TLR4 inhibitor (CLI‐095, Invivogen) or 10 mg/mL PI3Kinase inhibitor (LY294002, Invivogen). At 24 h, secreted CCL2 was measured in culture supernatant using Luminex Bead technology according to Bioplex protocols and cytokine kit (Biorad).

### Immunofluorescent microscopy

Tissues were snap‐frozen in OCT (Sakura) and stored at −80°C. Note that 7 µm sections were cut, air dried for 30 min, fixed in chilled acetone for 20 min, followed by 2× PBS washes. Sections were stained with primary antibodies (Table 1) for 20–30 min at room temperature, followed by 2× 5‐min washes in PBS. Secondary antibodies were added for 20–30 min. Slides were washed twice in PBS prior to mounting (ProLong Gold anti‐fade, Thermo Fisher) and imaged on a Zeiss LSM 800 confocal scanning microscope.

**Table 1:** Primary antibodies used.

Specificity	Antigen	Label	Clone	Manufacturer
Anti‐human	CD45	PE, PE‐Cy7	HI30	Biolegend
	CD3	BV421	UCHT1	Biolegend
	CD14	AF700	HCD14	Biolegend
	CD16	AF488, BV785	OKT4	Biolegend
	CD19	PerCP, BV711, AF594	HIB19	Biolegend
	CD56	APC‐Cy7, PE‐Cy7	HCD56	Biolegend
	CD64	APC	10.1	Biolegend
	CD135	BV711, APC‐Cy7	EH12.2H7	Biolegend
	CD163	AF647	RM3/1	Biolegend
	CD206	AF488, PE	15‐2	Biolegend
	CSF1	Unconjugated	AB233387	Abcam
	Collagen VI	Unconjugated	ERTR7	In house
	HLA‐DR	BV510	L243	Biolegend
	IL‐34	Unconjugated	AB101443	Abcam
Anti‐mouse	Podoplanin	PE‐Cy5, PE, APC	8.1.1	Biolegend
	CD45	PerCPCy5.5, PE, BV711	30‐F11	Biolegend
	CD45	PerCPCy5.5	I3/2.3	Biolegend
	CD31	Biotin	MEC13.3	Biolegend
	CD31	FITC	390	BD
	CD31	BV605, PECy7	390	Biolegend
	MERTK	Biotin	BAF591	R&D Systems
	MHCII	BV510, Pacific blue	M5/114.15.2	Biolegend
	CD169	FITC	3D6.112	Biolegend
	GR‐1	PE	RB6‐8C5	BD
	F480	PE, PE‐Cy5	Cl:A3‐1	Biolegend
	F480	PE	BM8	Biolegend
	Ly6C	FITC, PE‐Cy7, BV510	HK1.4	Biolegend
	Ly6G	PE	1A8	Biolegend
	CD11b	APC	M1/70	BD
	CD11b	APC‐Cy7, Unconjugated	M1/70	Biolegend
	Lyve‐1	Biotin	ALY7	eBioscience
	CD3e	Biotin	145‐2C11	BD
	CD3e	PerCPCy5.5	145‐2C11	Biolegend
	B220	Biotin	RA3‐6B2	BD
	B220	PerCPCy5.5	RA3‐6B2	Biolegend
	NK1.1	Biotin	MAb11	BD
	NK1.1	PerCPCy5.5	PK136	Biolegend
	CD135	Biotin	A2F10	BD
	CD135	PECy7	BV10A4H2	Biolegend
	CD11c	AF700	N418	BD
	Collagen VI	Unconjugated	ERTR7	In house

### Cell morphology analysis

The perimeter and area of cells were calculated using ImageJ. To ensure cells were seen in cross‐section, only those showing a DAPI^+^ nucleus were chosen for measurement. The morphology index is calculated as perimeter^2^/4πArea, where 1 is a circle, and values increase as a cell deviates from circularity [34].

### FRC and monocyte co‐cultures

Human FRCs at passage 3 were plated overnight at 2 × 10^4^ cells/0.32 cm^2^ well. The following day, 4 × 10^5^ human PBMCs were isolated from healthy donors using Ficoll‐Paque or Lymphoprep according to the manufacturer's instructions and added to appropriate wells. Where indicated, 0.1 µg/mL human anti‐CSF1R (Clone 61701, R&D systems) or isotype control (Clone 11711, R&D systems) was added. Cells were incubated for 72 h at 37°C, then harvested, quantified, labeled with antibodies (Table 1), and analyzed by flow cytometry (Supporting information Fig. S3C). For mouse assays, 2 × 10^5^ mouse FRCs were left to adhere to six‐well plates overnight. The next day 1 × 10^6^ mouse BM cells were added to each well, with 100 U/mL recombinant CSF1 (Peprotech) or 10 µg/mL purified anti‐mouse CSF1R/CD115 blocking antibody (Clone AFS98, eBioscience). Cells were harvested after 4 days, quantified using a Z2 Coulter Counter (Beckman Coulter, USA), labelled for flow cytometry, and then analyzed on a FACS Canto (BD Biosciences) using Flowlogic software version 1.7 (Inivai Technologies) or Flowjo software v10 BD Biosciences (Supporting information Fig. S3A).

### Statistical analysis

Normality was assessed using Shapiro–Wilk test. One‐way ANOVA with Tukey's posttest was used to compare three or more groups of parametric data. A *t*‐test was used to compare two groups of parametric data. Mann–Whitney *U* test was used for two groups of nonparametric data. Note that *p* < 0.05 was considered significant. For fold‐change data, a Wilcoxon rank test with a ratio paired *t* test was used. KEGG pathway analysis used the top 15 upregulated genes with LPS treatment. Pathways shown yielded *p* values < 0.05, with FDR and Benjamini–Hochberg values <0.05).

## Conflict of interest disclosure

The authors declare no commercial or financial conflicts of interest.

## Author contributions

J.D.R. designed the study, performed experiments, analyzed data, and wrote the paper. A.L.F. and T.S.P.H. designed the study, analyzed data, wrote the paper, and secured funding. K.K., H.W.C., Y.A., J.D.D.C., J.A., and E.D. performed experiments and analyzed data. M.L., C.P.S., and D.R. performed and analyzed experiments. A.D., S.J.T., and R.B. provided essential reagents and guidance. M.S. designed experiments. S.N.M. and B.L. provided essential reagents and designed and analyzed experiments. All authors read and approved the manuscript.

## Ethics approval

Palatine tonsils and healthy blood were obtained from the National Disease Research Interchange resource center, the HBRC, Birmingham (HTA licence 12358, 15/NW/0079, project number REC/RG/HBRC/12‐071) or Monash University (MUHREC), project number 11939, all with written informed consent. All tissues were obtained and utilised in accordance with institutional guidelines and according to the principles expressed in the Declaration of Helsinki. Research utilizing animals was performed at University of Melbourne, and in accordance with state, federal, and cantonal guidelines (Tierschutzgesetz) under permission numbers SG07/19 following review and approval by the Cantonal Veterinary Office (St. Gallen, Switzerland) or with approval from University of Melbourne Animal Ethics Committee. Where permitted, tissues for in vitro work were obtained from mice euthanized for other projects as an exempt use in accordance with principles of the 3 R's. All mice were specific pathogen free and cared for in accordance with institutional guidelines. All animal experiments received approval from relevant institutional ethics committees.

**Abbreviations**
DTR: diphtheria toxin receptor
DTx: diphtheria toxin
FAP: fibroblast activation protein
FRC: fibroblastic reticular cell
HBRC: Human Biomaterials Resource Centre
LEC: lymphatic endothelial cell
TBRC: T‐B zone reticular cell
TRC: T‐zone reticular cell

## Supporting information

Supporting Information

## Data Availability

Data supporting the findings of this study will be provided upon reasonable request to the corresponding author. Human RNA‐Seq data are accessible at monash.figshare.com doi: 10.4225/03/ 5a2dae0c9b455.
