# Supplementary material for: Fibroblastic reticular cells provide a supportive niche for lymph node–resident macrophages
Source: Eur J Immunol. 2023 Jul 12;53(9):2250355. doi: 10.1002/eji.202250355 (PMC10947543; doi:10.1002/eji.202250355)

Supplementary figure legends

Supplementary figure 1: Fibroblastic reticular cell depletion leads to a reduction in innate cell populations. A. CCL19-DTR mice, or DTR-expressing Cre-negative littermate controls were treated with diphtheria toxin, and brachial lymph nodes were harvested and analysed by flow cytometry at 2, 8 or 22 days after treatment ceased. Gating strategy for FRCs, monocytes and macrophages is shown. **B.** FAP-DTR and non-transgenic littermate control mice were each given 25ng/g diphtheria toxin (DTx) on alternate days for 6 days prior to harvest. On day 6, lymph nodes were harvested and digested according to previous protocols and analysed via flow cytometry. Timeline of DTx administration and harvest. **C.** The flow cytometry gating strategy for lymph node FRCs, monocytes, macrophages and neutrophils is shown **D.** Cell counts for lymph node FRCs, macrophages, monocytes and neutrophils. Mean + SEM, n = 4 mice from one experiment. *P<0.05, ** P<0.01, T-test.

Supplementary figure 2: Single cell transcriptomic analysis of isolated EYFP+ reticular cells from murine lymph node stromal subsets during inflamed and resting states. $EYFP^+$ reticular cells were isolated from brachial lymph nodes from Ccl19 Cre R26R-EYFP mice, which were either treatment-naïve, or immunised with OVA/LPS. scRNA-seq was performed on $EYFP^+$ cells. UMAP of $EYFP^+$ lymph node reticular cell subsets, categorised into 8 subsets, with or without treatment. **A, B:** Histograms of isolated EYFP+ reticular clusters showing **A.** Absolute and **B.** relative abundance of LPS treated and naïve analysed cells per reticular cell cluster. **C**. Heatmap of curated marker gene expression for identified reticular subsets. **D**. KEGG pathway analysis of genes upregulated with LPS treatment (*P*<0.05 depicted with dotted line, FDR and Benjamini-Hochberg <0.05). **E.** Violin plots showing expression of Ccl2 by FRC subsets in treated or naïve mice. **F.** Human lymph node or tonsil FRCs from 3 donors were cultured *in vitro* and stimulated with 1μg/ml of LPS for 24h, with CCL2 protein measured using Luminex Bead technology. Fold-change from untreated cells is depicted. Mean + SEM shown, n = 3 individual human donors from 2 independent experiments. Mean + SEM shown, n = 3 individual human donors from 2 independent experiments. * p<0.05, one-way ANOVA with Tukey's multiple comparison test, comparing to untreated. PI3Kinh = PI3K inhibitor; TLR4inh = TLR4 inhibitor.

Supplementary figure 3: Fibroblastic reticular cells support monocyte differentiation via CSF1R signalling. $1x10^6$ mouse bone marrow cells, as a source of macrophage precursors, were co-cultured with $2x10^5$ mouse FRCs under various conditions. Cells were harvested and quantified after 3 days, and assessed using flow cytometry. **A.** Monocyte and macrophage gating strategy for flow cytometry. **B.** Macrophage numbers after 3 days of co- culture with or without recombinant CSF1 and CSF1 blocking antibody, performed in triplicate, from one experiment. **** P<0.0001, one-way ANOVA with Tukey's post-test. **C.** $4x10^5$ human peripheral blood mononuclear cells (PBMCs) were phenotyped immediately after isolation (0H) or incubated with or without LPS, CSF1R blocking antibody, isotype control antibody, or $2x10^4$ human tonsil-derived FRCs. After 72 hours of culture, cells were quantified and analysed via flow cytometry. The gating strategy is shown for M1 and M2 macrophages, and classical and non-classical monocytes, related to data from Figure 4C-H.

Supplementary Figure 1

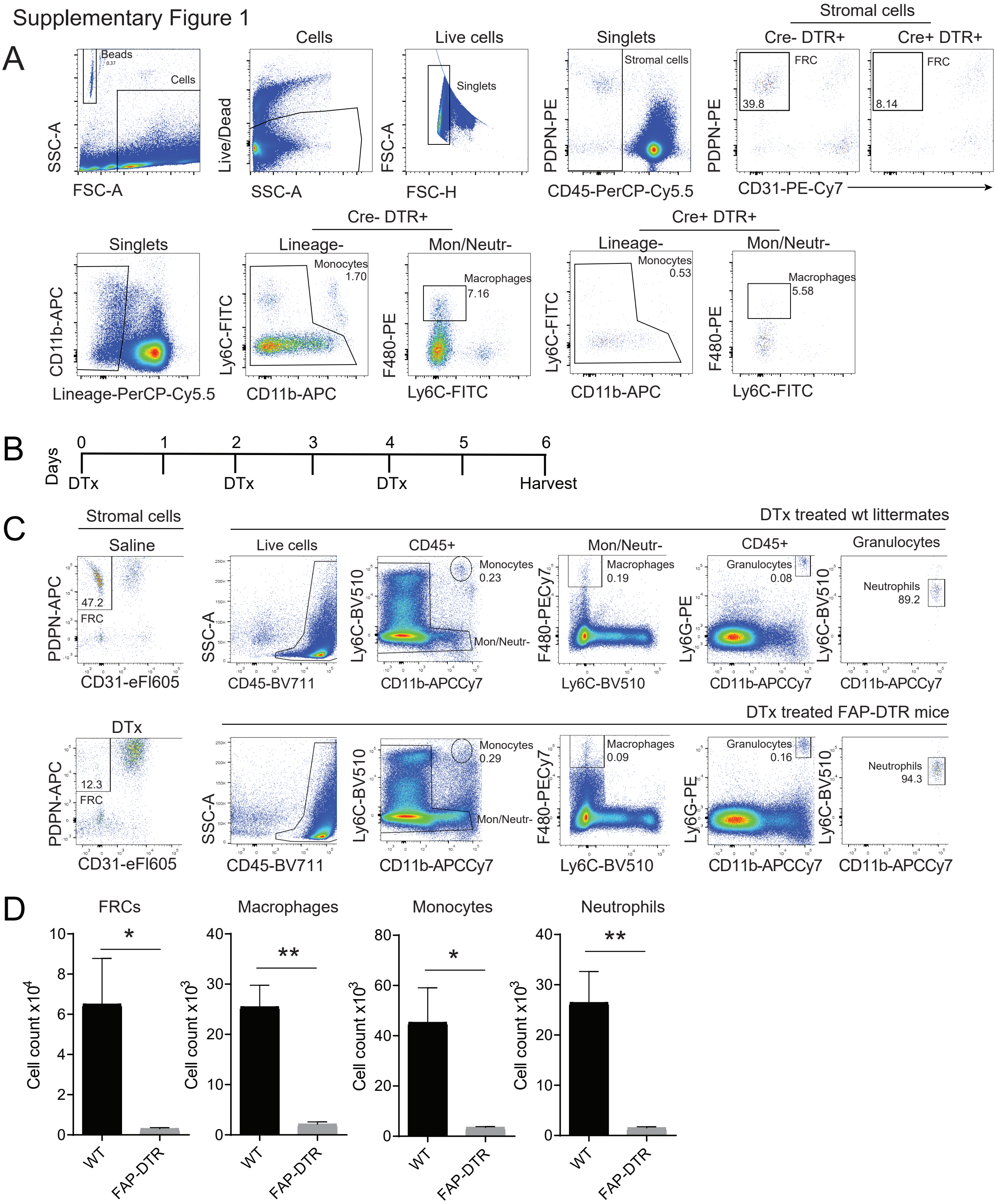

Supp. Figure 2 (related to Figure 3)

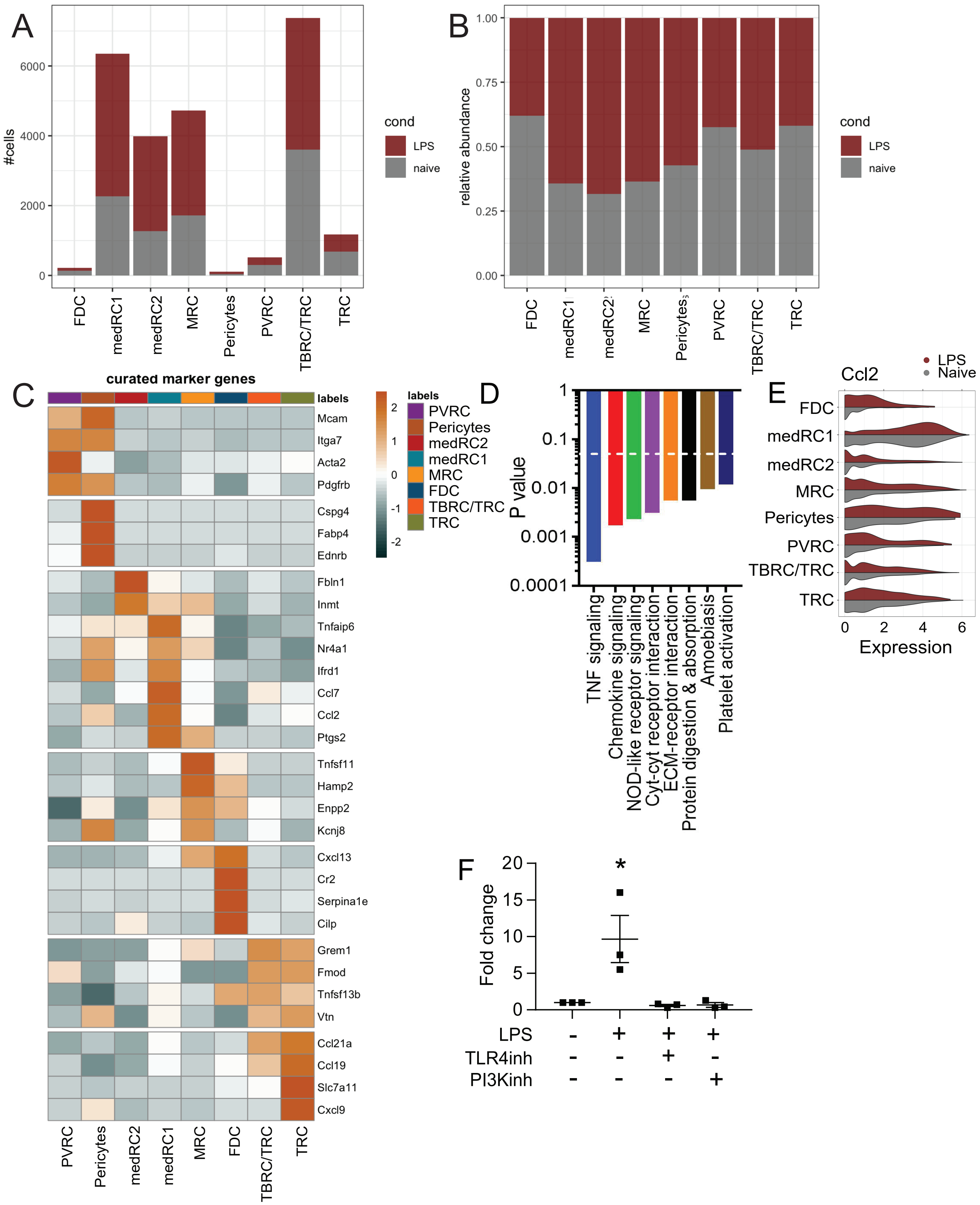

Supp. figure 3 (related to Figure 4)

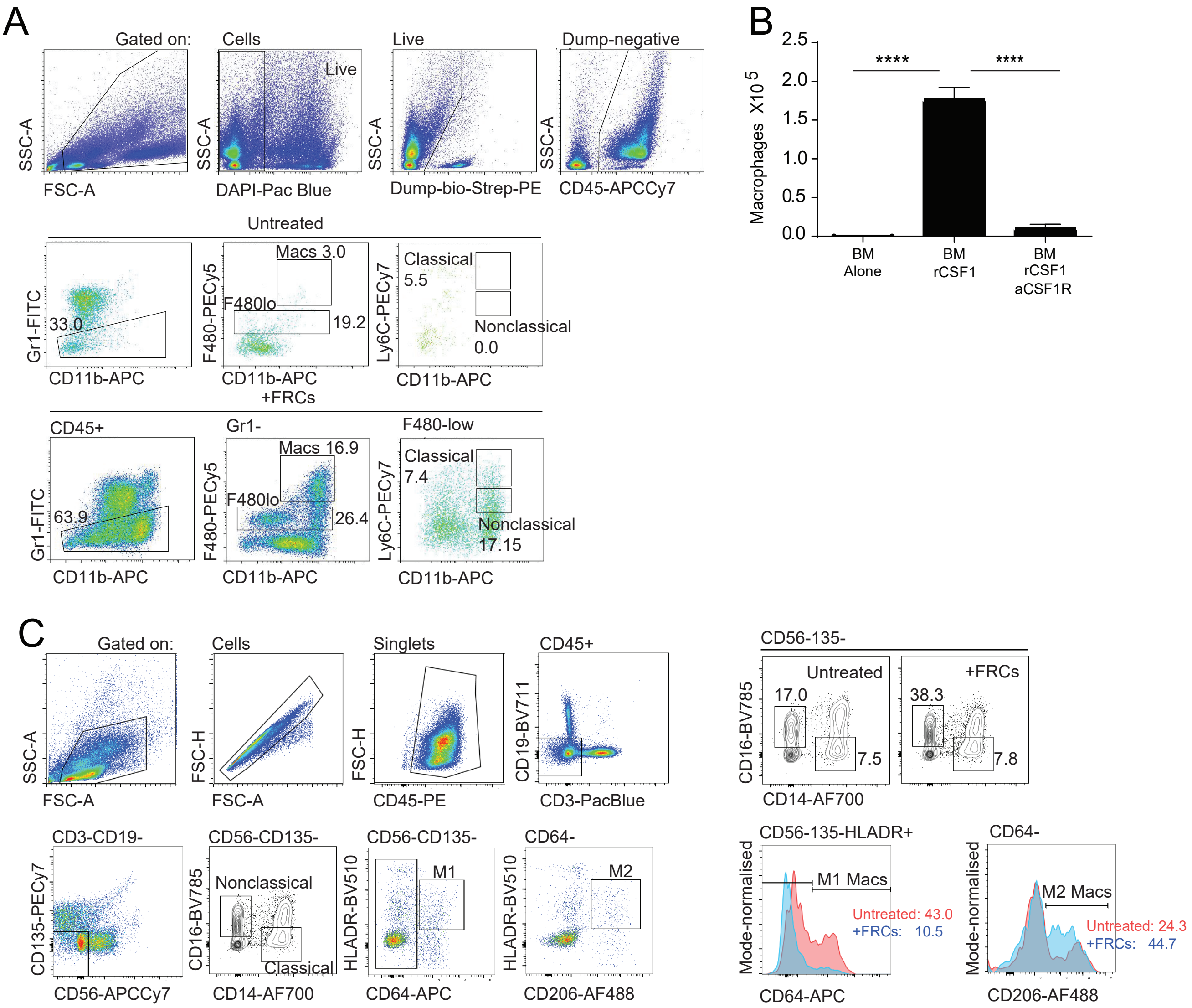

Supplement: Supplementary file 1 — Supporting Information [file EJI-53-0-s001.pdf]
